# The Features and Functions of Neuronal Assemblies: Possible Dependency on Mechanisms beyond Synaptic Transmission

**DOI:** 10.3389/fncir.2016.00114

**Published:** 2017-01-10

**Authors:** Antoine-Scott Badin, Francesco Fermani, Susan A. Greenfield

**Affiliations:** ^1^Neuro-Bio Ltd., Culham Science CentreAbingdon, UK; ^2^Department of Physiology, Anatomy and Genetics, Mann Group, University of OxfordOxford, UK

**Keywords:** neuronal assemblies, synaptic transmission, volume transmission, gap junctions

## Abstract

“Neuronal assemblies” are defined here as coalitions within the brain of millions of neurons extending in space up to 1–2 mm, and lasting for hundreds of milliseconds: as such they could potentially link bottom-up, micro-scale with top-down, macro-scale events. The perspective first compares the features *in vitro* versus *in vivo* of this underappreciated “meso-scale” level of brain processing, secondly considers the various diverse functions in which assemblies may play a pivotal part, and thirdly analyses whether the surprisingly spatially extensive and prolonged temporal properties of assemblies can be described exclusively in terms of classic synaptic transmission or whether additional, different types of signaling systems are likely to operate. Based on our own voltage-sensitive dye imaging (VSDI) data acquired *in vitro* we show how restriction to only one signaling process, i.e., synaptic transmission, is unlikely to be adequate for modeling the full profile of assemblies. Based on observations from VSDI with its protracted spatio-temporal scales, we suggest that two other, distinct processes are likely to play a significant role in assembly dynamics: “volume” transmission (the passive diffusion of diverse bioactive transmitters, hormones, and modulators), as well as electrotonic spread via gap junctions. We hypothesize that a combination of all three processes has the greatest potential for deriving a realistic model of assemblies and hence elucidating the various complex brain functions that they may mediate.

## Features of Neuronal Assemblies

“Neuronal assemblies” (Gerstein et al., 1989; Singer et al., 1997) are large scale coalitions of neurons that operate in collective activity over a spatio-temporal scales of millimeters and milliseconds, i.e., at “meso-scale” level of brain organization thereby linking cellular (micro-scale) and entire neuronal systems (macro-scale) events (Eytan and Marom, 2006). However, this definition is not universally accepted: the same term has been used for different, yet sometimes overlapping entities, such as anatomically defined cortical columns (Krueger et al., 2008): conversely the same phenomenon featured here, namely the *dynamic* patterns generated by thousands and tens of thousands of co-active cells, has been referred to in alternative terms, such as “ensembles” (Pais-Vieira et al., 2013; Miller et al., 2014; Carrillo-Reid et al., 2015).

If the defining feature then, is one of dynamism, then one of the most effective ways of studying assemblies is with molecular probes, which anchor within fatty environments of neuronal cell membranes, and are sensitive to real-time changes in membrane electrical potentials (*V*_m_) (Loew, 1996). Voltage-sensitive dyes (VSDs), which possess electrochromic properties that enable them to report ongoing electrical potential changes (Grinvald et al., 1982), such as di-4-ANEPPS (Tominaga et al., 2000; Petersen et al., 2003a,b), are particularly effective. Di-4-ANEPPS is one of the most useful and versatile VSDs – it is a red dye which produces data with relatively high signal-to-noise ratio and more commonly used *in vitro* (Badin et al., 2013, 2016; Preuss and Stein, 2013; Gandolfi et al., 2015), which has been implicated as a less toxic alternative to blue dyes (themselves originally developed for *in vivo* applications) and which can also be used *in vivo* (Grandy et al., 2012). Using mathematical analysis software, such as MatLab (Mathworks) or Mathematica (Wolfram), three-dimensional voltage-sensitive dye imaging (VSDI) data sets (fluorescence × space × time) can be processed in any way, shape or form using bespoke analysis scripts and codes. Whilst both electrophysiology and VSDI offer high temporal (millisecond) resolution, only the latter reveals spatial features (micrometers). For example, whilst the spread, time course and amplitude of optical activity signals are among the most popular parameters for describing assemblies, other less obvious yet appropriate measurements for the particular experiment at hand have emerged, such as “summed overall fluorescence” (Badin et al., 2016), “Time-to peak” (Hill and Greenfield, 2014; Gandolfi et al., 2015) or population activity propagation speed (Yuste et al., 1997; Tominaga et al., 2000), to name but a few. Though it remains difficult to unequivocally attribute specific physiological meaning to each of these parameters, they still reflect the summed output of veritable dynamics of population activity.

Assemblies will to some extent feature specific spatio-temporal profiles determined by the network-specific cytoarchitecture of particular brain regions: for example, fast, low amplitude responses are typical of sub-cortical relay structures (such as the thalamus and basal forebrain) compared to those of cortex, which are comparatively more extensive in time and space (Badin et al., 2016). However, additionally to the specific physical network cytoarchitecture, these characteristics can also be much influenced by the experimental preparation and protocol. There is an inevitable trade-off between investigation of assemblies under more holistic and physiological conditions (such as those seen *in vivo*) compared to the reductionist, albeit more controlled scenario of limited neuronal connectivity, as seen *in vitro*, which nonetheless gives direct access to brain regions other than cortex: the approaches are complementary and equally necessary.

*In vitro* experiments are performed on either slices cut in the coronal plane, or in a parasagittal section (Agmon and Connors, 1991) preserving thalamo-cortex connectivity: in either case the full depth of the cortex can be investigated, whereas *in vivo* experiments focus on a top-down dorsal visualization (i.e., looking down onto the pial surface of the cortex once the skull has been removed), where there is an inevitably greater focus on superficial layers. More specifically: *in vivo* protocols reveal that the blue dye RH-1619 penetrates 350–400 μm into the depth of the cortex from the pial surface, after a 2 h-long staining period, providing information on activity within layers I and II/III (Petersen et al., 2003a). By comparison, for *in vitro* experiments, VSDs have been reported to penetrate approximately 100 μm within slice tissue after 30 min-long staining periods using the red dye di-4-ANEPPS (Hill and Greenfield, 2014), providing fluorescence information originating from sufficient volumes of neuronal tissue in all layers.

An important and general factor could be that the slice preparation removes any influence of more global signaling systems: destruction of the overall organization of the brain and the inevitable disruption of all long-range connections, such as the diffuse monoaminergic ascending systems from midbrain/brainstem nuclei (Carter and Fibiger, 1977; Azmitia and Segal, 1978; Levitt and Moore, 1978), which will lead to a substantially reduced tonic neuromodulatory influence of signaling molecules, such as dopamine, noradrenaline, and serotonin. Compounding this lack of neuromodulatory influence, the existence of other neurotransmitter systems (other than monoaminergic) also implicated in neuromodulation of network activity via extra-synaptic receptor (tonic) activation (Nakanishi, 1992; Descarries and Mechawar, 2000; Belelli et al., 2009), a mechanism of volume transmission (Vizi et al., 2004), are also lost in slice preparations. Such mechanisms of neurotransmission have been implicated for acetylcholine (Descarries et al., 1997), glutamate (Huang, 1998; Min et al., 1998; Szapiro and Barbour, 2007), GABA (Kullmann, 2000; Mody, 2001; Olah et al., 2009) and many more less familiar messengers, such as hormones and neuropeptides (Kercel, 2004; Trueta and De-Miguel, 2012). Hence, the complex population dynamics reported *in vivo* will show much simplified profiles when recorded *in vitro*, where sub-cortical systems, long-range connectivity and extra-synaptic volume concentrations of various bioactive molecules will no longer play a decisive role in gating the full processing abilities of cortical networks.

For example, neuronal assemblies generated *in vivo* in various cortical areas (Bernasconi et al., 2000; Rokem et al., 2010; Pinto et al., 2013), show a similar overall activity as their *in vitro* counterparts, i.e., thalamocortical, somatosensory or visual cortical slices (Berger et al., 2007; Sato et al., 2012; Soma et al., 2012), yet retain more complex profiles, such as large depolarisations accompanied by inhibition that is both spatial (surround inhibition) and temporal (rebound hyperpolarisation), presumably in both cases to enhance the signal-to-noise ratio (Petersen et al., 2003a; Borgdorff et al., 2007; Devonshire et al., 2010b). This effect, however, can be abolished by deepening anesthesia, suggesting that it operates a significant physiological function in information processing. Such a notion is further supported by the fact that assemblies can also be dramatically modulated by systemic administration of other bioactive substances, from the silencing effects of anesthetics (Devonshire et al., 2010b; Hama et al., 2015) to the broad and erratic epileptiform activity induced by agents such as gabazine or bicuculline (Lippert et al., 2007). Moreover, assemblies can become less extensive, in response to identical stimuli, in adult compared with juvenile animals (Badin et al., 2016), further suggesting assemblies are highly dependent on context-specific factors and play a part in on-going functions.

## Functions of Neuronal Assemblies

Drawing on data from both *in vitro* and *in vivo* studies, a wide range of brain functions can now be better understood by reference to assemblies (von Stein and Sarnthein, 2000; Buzsaki and Draguhn, 2004), from visual processing (Vucinic and Sejnowski, 2007; Greenberg et al., 2008; Miller et al., 2014) to impact of depth of anesthesia on evoked sensory responses (Devonshire et al., 2010b), impact of learning-induced plasticity on assembly size and dynamics (Devonshire et al., 2010a), as well as revealing previously unappreciated but basic differences between analgesics, (morphine and gabapentin), and anesthetics, (thiopental and propofol) (Collins et al., 2007).

Yet whilst known functions can be more accurately described in terms of activity patterns, assemblies themselves might be a good starting point for understanding previously elusive functions. Their emergent spatio-temporal profile typically is one of hundreds of milliseconds, a time-course roughly three orders of magnitude greater than the action potential which trigger them – between 0.2 and 0.7 ms (Borst et al., 1995; Gray and McCormick, 1996): this collective, network-wide output could correspond to one-off, unique brain states, such as eventually a moment of consciousness, for the following reasons. First, neural activity only appears to contribute to a state of consciousness when it is continuous and sustained (Bachmann, 2000): the observed time-windows of activity, lasting several hundred milliseconds, are found to coincide with the time taken for conscious perception of stimuli; which occurs at the crucial threshold of 270 ms (Vogel et al., 1998; Sergent et al., 2005). Secondly anesthetics, which by definition abolish consciousness, significantly retard specific parameters of individual assembly dynamics (such as peak width and termination of activity) both *in vitro* (Collins et al., 2007) and *in vivo* (Devonshire et al., 2010b). Thirdly, a time window of approximately this length demarcates the earliest spatial differentiation of distinct patterns in assemblies for subjective differentiation of sensory modalities (Chakraborty et al., 2007). Fourthly, the energy will need to be conserved in some chemical, electrical, or thermal form (Blundell and Blundell, 2009). In the case of heat, pressure in the neuronal micro-environment will increase, and vice versa: perhaps this could explain why increased pressure and hence an increase in thermal energy, will lead to both the onset of consciousness in anesthetized animals (Kendig et al., 1988) as well as a significant increase in assembly size (Wlodarczyk et al., 2006). Taken individually, these arguments are each relatively weak for linking neuronal assembly function to consciousness. However, we believe that these findings represent interesting coincidences, which, collectively hint at a possible link.

Rather than single unit activity and isolated synaptic signaling, it is neuronal assemblies that perhaps can be more accurately regarded as the building blocks of the central nervous system (Rinkus, 2010; Beul and Hilgetag, 2014; Miller et al., 2014): they provide the all-important link enabling bottom-up cellular events to be realized as top-down functions. Yet little is known about how such translation is possible. A first step will be to understand the mechanisms responsible for the generation and propagation of assemblies themselves; which drive them to spread more extensively in both space and time, thereby granting them much greater informational receptive fields as well as greater time-windows for integration of information and processing, compared to that which traditional neuronal signaling would otherwise permit.

## Underlying Mechanisms Governing Assembly Dynamics

### Synaptic Transmission

Synaptic transmission is the well-known signature of neuronal signaling with time-frames of information transfer down axons of 0.1–100 m/s, and time-delays for information to cross synapses (at 38°C) of around 150–300 μs (Sabatini and Regehr, 1999). However, some anomalies become immediately evident when comparing assemblies generated in two different, well-established *in vitro* preparations: first, coronal brain slices where assemblies are evoked using direct electrical stimulation to the cortex (Yuste et al., 1997; Zochowski et al., 2000; Petersen and Sakmann, 2001; Bourgeois et al., 2014), and secondly, a thalamocortical section (Agmon and Connors, 1991; Takashima et al., 2001; Llinas et al., 2002; Hill and Greenfield, 2014) that enables indirect, remote activation of the cortex region via neuronal innervation resulting from thalamic stimulation. Other studies have also investigated the downstream cortico-cortical connectivity elicited by exogenous activation of the lemniscal pathway, leading for example to active communication between primary somatosensory cortex and motor cortex (Ferezou et al., 2007) or for the purposes of general brain mapping *in vivo* (Lim et al., 2012).

**FIGURE 1:**
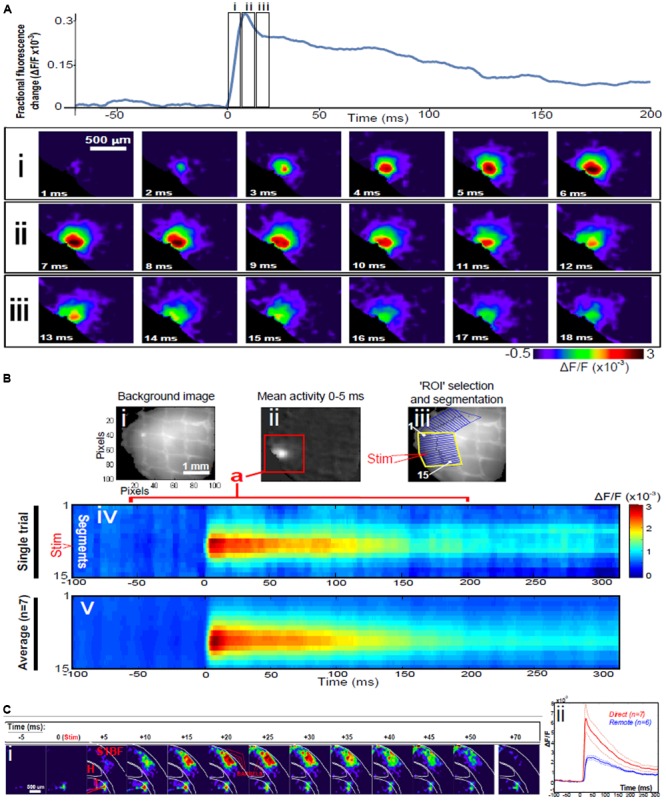
**Dynamics of assemblies evoked in direct and remote activation. (A)** Fluorescence time-series from a representative experiment where a 30 V electrical pulse (0.1 ms in duration) was delivered to Layer II/III of agranular insular cortex (AIC, part of prefrontal cortex – PFC), focusing on peak response of evoked neuronal assemblies; this experiment was repeated seven times, each using slices from different rats. Still-frame sequential activation of an assembly showing the very fast rise (0–6 ms, **i**), followed by the peak fluorescence emission (6–8 ms, **ii**) and a portion of the slow decay back to baseline (8–200+ ms, **iii**). Refer to Supplementary Materials for all methods of dissection, slicing, VSD staining and imaging and data analysis. **(B)** Image from the slice, cut in a coronal plane, of PFC containing AIC in the recording bath, as seen through the voltage-sensitive dye imaging (VSDI) optics (**i**), raw data average of fluorescence between 0 and 5 ms after stimulation (**ii**) and selection of the region of interest (ROI) and its segmentation for analysis (**iii**, yellow area represents the ROI selected): binning of data and representation into colour-coded arrays, or “space-time” maps (**iv**). Space-time maps: segments 1–15 in **iii** are graphed on the *y*-axis, against time (milliseconds, *x*-axis, **iv**). Single experiment data sets (as in **iv**) can then be combined to produce overall experiment averaged space-time maps (**v**, *n* = 7). Image processing via toolbox of Bourgeois et al. (2014). Red box in **B ii** represents the imaging area of focus in **A i**–**iii**, while the time-span highlighted in red in **B iv** represents the epoch graphed out as a time-series in **A**. Thalamocortical slice: **C**. Still-frame panels showing the sequential activation of barrel field cortex (**i**) in response to a single 60 V stimulation pulse to the Thalamus (VPM nucleus). Individual barrels can be seen to activate, with a higher signal intensity (black), within the cortical mantle, in particular within the still-frames +20 and +25 milliseconds (ms, **i**) after stimulus delivery. Here it can be seen that the stimulus delivered to the thalamus at *t* = 0 takes less than 5 ms to reach and start activating Layer IV barrels within the cortex, however then it takes another 20 ms to fully activate the cortex. Inset graph (**ii**) shows fluorescence readings measured over time, from both direct (as in **A, B**) and remote **(C)** stimulations in red and blue time-series, respectively, including SEMs (thin dotted lines), showing the great disparity and distinct activation profiles. Labels: H, hyppocampus; T, thalamus; S1BF, primary somatosensory barrel field cortex.

**FIGURE 2:**
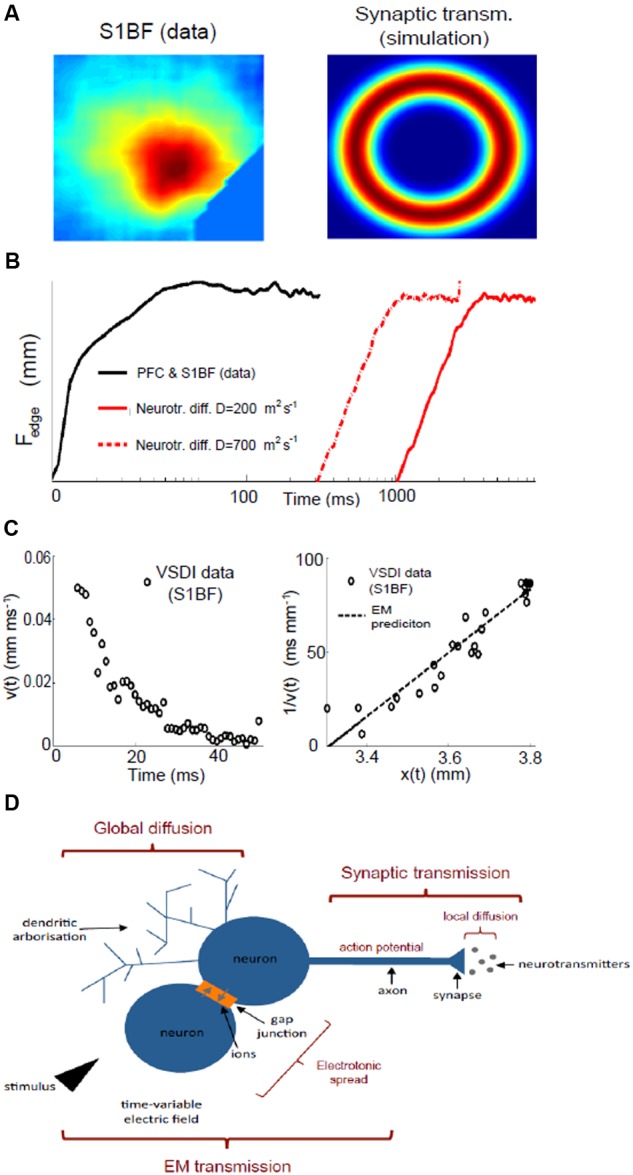
**The need for three fundamental mechanisms underlying neuronal assemblies dynamics. (A)** Evoked activity in the somatosensory barrel field cortex (S1BF) measured with VSDI (left) compared with a simulation of a two-dimensional network of spiking neurons subject to the same central stimulus, which is applied for a very short duration (0.1 ms), and where activity spreads due to synaptic transmission alone. Refer to Supplementary Materials for all methods of data modeling. **(B)** Comparison of assembly spread between PFC and S1BF data (PFC and somatosensory – black line) and simulation with passive diffusion only (red lines): the diffusion profile from simulations can be considered an upper edge for diffusion speed: we simulated 5 × 10^5^ neurotransmitters released at position 0 and considered half a dozen neurons surrounding the releasing origin that acted like sinks; in reality there are far more neighboring neurons to up-take the neurotransmitters so that diffusion is bound to be slower than what estimated with this proof-of-concept experiment. **(C)** Spatial and velocity profile of an assembly in somatosensory (S1BF) cortex: we plot the rate of spread (velocity) of an assembly as a function of time (left) and its inverse against space (right), which we compare with the electromagnetic (EM) prediction (see text). **(D)** Dendrites (top left) can release bioactive agents independent of action potential generation, and diffuse broadly affecting sub-threshold dynamics. Synaptic transmission (top right) is the process by which a neuron releases an action potential after the electrochemical gradient between inside and outside the cell has been inverted by incoming currents. At the synapse, the action potential triggers the diffusion of neurotransmitters, which open ion channels in the post-synaptic dendrites. Gap junctions are a direct electrical link between two cells and allow ions currents. Together with the time variability of the stimulus, electrotonic spread favors the onset of self-sustained electromagnetic waves (bottom), which excite neighboring neurons faster and more widely than synaptic transmission alone.

Direct stimulation with a single electrical pulse evokes activity from an epicenter with fast and efficient recruitment of large numbers of neurons in near-synchronous fashion manifesting as a circular propagating wave of activity spreading outwards from the locus of stimulation (Lopes da Silva, 1991) as seen using VSDI, **Figure 1A**. This is a stereotypical activation pattern which has been reported in virtually all studied neuronal population systems using VSDI, both in the cortex and sub-cortical structures (Badin et al., 2016), and it is those dynamics which can be modulated with bioactive compounds. Signal propagation via action potentials traveling down axons and activating chemical transmitters at synapses with the neurons it contacts takes just over 1 ms. The speed of action potential propagation varies significantly across circuits in the brain as well as with the distance they travel, but even the slowest signals (traveling through unmyelinated axons) take 0.5 ms to travel 1 mm, while subsequent transmitter release and diffusion across the synaptic cleft is approximated to take just under 0.75 ms. The activation of synapses has been found to decay with time-scales that go from a few ms (e.g., for synapses rich in GABA_A_ and/or AMPA receptors) to 100 ms (for those most influenced by GABA_B_ or NMDA receptors). However, if this was the dominant mechanism at play, given the speed of transmission, the greatest activity would most probably be observed furthest from where the stimulus was received, i.e., at the spreading perimeter (**Figure 2A**), a configuration which conflicts with the data. Under normal conditions in direct stimulation paradigms, assemblies usually reach maximum lateral spread within 5–6 ms after stimulus delivery (**Figure 1B**), while by comparison, for remote thalamocortical stimulation (i.e., neuronal activation of cortical tissue), this occurs between 10 and 13 ms after stimulation, as seen in **Figure 1C**, is delivered to the cortex (i.e., with time delay corrected for impulse conduction time from thalamus to cortex – of the order of 4–5 ms) (Landisman and Connors, 2007); i.e., where assemblies are evoked in a more physiological manner than those triggered with direct electrical stimulation. This scenario suggests that other factors, in addition to traditional synaptic transmission, may be affected by this difference in stimulation paradigm; leading to the emergence of different profiles of activation dynamics and resulting time courses.

In terms of *time*, it takes some 300 ms for decay of assembly activity to fall to even 20% of its maximum strength (Chakraborty et al., 2007). The dye used to visualize voltage changes has a latency and decay time of the order of a millisecond or less (Tominaga et al., 2000): hence, the persistent activity observed must be a genuine physiological phenomenon. This continuation of activity over several hundred milliseconds could be attributable to the prolonged duration of signal decay time-scale of synapse operations that can last up to 100 ms (Hestrin et al., 1990). However, if synaptic transmission were the sole mechanism, the greatest activity would be observed furthest from where the stimulus was received, i.e., at the spreading perimeter (**Figure 2A**): yet this configuration conflicts with the experimental data (**Figure 1**). Alternatively, the typically lengthy time frame of an assembly could represent the summation of thousands of sequential synaptic connections whereby the cumbersome process of neurotransmission will impede the signal speed. However, this scenario too can potentially be discounted if *spatial* features are now considered. There are two possible scenarios which could account for the characteristic and extensive assembly spatial spread. One option is that, most probably at the locus of stimulation, a small proportion of directly activated neurons fire action potentials, which in turn propagate potentials affecting the resting membrane potential of target neurons with which they form synapses. In this case, the length-scale of interaction will be a function of synaptic connectivity, network coupling strength (balance between inhibition and excitation) and synaptic dynamics where the firing of a neuron at any given point will affect, above the reference threshold, neurons about 50% further than the connectivity length-scale (Jirsa and Haken, 1996). Typically (Perin et al., 2011), this metric implies a spatial spread that will still be, nonetheless, two to five times less than actually observed, and can thus be discounted.

The second and more plausible option as the underlying dominant process, and one in any case that is preferentially detectable with voltage sensitive dyes (Chen et al., 2012): a widespread sub-threshold depolarisation. If so, a second signaling mechanism is needed that could accommodate the extensive spatial spread. Just such a mechanism is also suggested by a discrepancy between predictions from synaptic transmission alone, and what is empirically observed in time frames (**Figure 1C**): whilst the transmission of a signal from thalamus to cortex, via classic synaptic transmission, takes only 5 ms to travel some 1–2 mm, a further 20 ms is required for the assembly to spread within the cortex, to its full extent.

### Volume Transmission

Volume transmission enables interaction between neurons in a way that is much less specific and significantly slower, yet with the pay-off that it involves far more cells at any one time: it is considered a complementary counterpart to classic synaptic “wired” transmission (Agnati and Fuxe, 2014; Taber and Hurley, 2014). In fact, it has been known since the 1970s that classic transmitters such as dopamine can be released (Nedergaard et al., 1989) as can protein (Greenfield et al., 1980) from a part of the neuron dendrites that typically has a very different role. Normally, dendrites, traditionally regarded as being the target for incoming connections, can actually release substances in their own right, and do so independent of the action potentials generated at the cell body (Greenfield, 1985). Moreover, this dendritic release also affects a much wider area than standard synaptic transmission, is far less precise and uses different ionic mechanisms and cellular storage, – all suggesting a contrasting yet complementary modulatory process. In addition to neuro-active chemical release from dendrites, there are a wide range of afferent fibers originating from sub-cortical structures, specialized in releasing neuromodulatory chemicals, such as acetylcholine, dopamine, noradrenaline, and serotonin (to name but a few), which do not form traditional synapses, but instead are specialized in releasing large amounts of modulatory transmitters into the extracellular space for tonic influence of population activity (Seguela et al., 1987; Soghomonian et al., 1989; Descarries and Mechawar, 2008). Every neurotransmitter system potentially influences neuronal networks in a tonic manner (Zoli et al., 1999; Kullmann, 2000), i.e., via volume transmission, as would the presence of extra-synaptic glutamatergic and GABAergic receptors suggest (Belelli et al., 2009), and as has already been widely reported for monoaminergic and cholinergic systems (Beaudet and Descarries, 1978; Descarries and Mechawar, 2000). In addition to these well known transmitters, a wide range of other endogenous signaling molecules act exclusively via volume transmission: growth factors (Carro et al., 2000), hormones (Mody, 2008), and peptides (de Wied et al., 1993), confirming that volume transmission is an essential and effective mechanism of cell signaling, operating on both high time- and spatial-scales. But whilst synaptic transmission is too local and too fast, the passive diffusion of bioactive agents beyond the synapse, volume transmission, is too slow a mechanism for the generation of assemblies (**Figure 2B**), In order to counterbalance the slow speed of the extensive extra-synaptic outreach of volume transmission, what is required now to accomodate the characteristic dynamics of an assembly, is an ultra fast signaling system (Steriade, 2005).

### Electromagnetic Transmission

Electrotonic spread via gap junctions (Anava et al., 2013; Agnati and Fuxe, 2014) is a widespread mode of close-range and high-speed neuronal signaling and has been reported to exist both in inhibitory and excitatory networks of coupled neurons (Tamas et al., 2000; Traub et al., 2001a). Gap junctions are a form of intercellular connection, where the trans-membrane pores formed by a congregation of connexin proteins in the membrane of two neighboring cells creates an open channel, such that the cytoplasm of two cells are effectively continuous, allowing free flow of ions and therefore: electrical pulses. In addition to neurons, connexin proteins are expressed in a range of cells involved in neuronal communication, including glial cells, where gap junctions also mediate substantial parts of their communication (Nagy and Rash, 2000; Orthmann-Murphy et al., 2008), allowing them to carry out essential roles in the maintenance of network health for appropriate functioning (Watkins and Maier, 2002). These low resistance connections have been mapped throughout the brain (Nagy et al., 2001), highlighting their ubiquitous expression and function throughout the CNS in inhibitory (Beierlein et al., 2000; Tamas et al., 2000; Krook-Magnuson and Huntsman, 2005; Fukuda et al., 2006) and excitatory neurons (Spray and Bennett, 1985; Traub et al., 2001b). Gap junctions have been found to play a key role in operating a range of functions including neuronal differentiation (Bani-Yaghoub et al., 1999), synaptogenesis and circuit formation (Connors et al., 1983; Peinado et al., 1993; Kandler and Katz, 1995), yet the role for which they are considered here as a key method of cell signaling in assembly generation is for their permissive capacity in transmembrane impulse propagation (Spray and Bennett, 1985; Bennett, 1997), allowing signaling independent of transmitter release (Eugenin et al., 2012; Leybaert and Sanderson, 2012; Belousov and Fontes, 2014). In accordance with these findings, it has been found that in neuronal networks, very fast oscillations (200 Hz), that could underlie the sustained activity seen here in assemblies, are mediated not via synapses but by these gap junctions (Draguhn et al., 1998). So if fast oscillations underlie the sustained activity seen here in assemblies (van den Pol, 2012), although they will take longer to effectively reach a maximum, once underway (Petersen et al., 2003a), the spread of activity will reach further than any signal from a synapse ever could. Hence, assemblies will be a very appropriate neuronal correlate for the space-time requirements for consciousness since, unlike localized neuronal circuitry, *they are neither hard-wired in time, nor spatially restricted*.

The existence of an electrochemical gradient across neuronal cell membranes generates a small electric field (Kandel et al., 2000), and the diffusion of ions contributes to the time-variability of this electric field, possibly representing *per se* a further form of signaling: electromagnetic transmission. Time-variable electric fields induce electromagnetic fields and vice versa (Jackson, 1962), thus allowing for electromagnetic energy to propagate directly via gap junctions and in the form of electromagnetic waves from the source of the signal, exciting neighboring neurons. Energy conservation will result in the intensity of the energy being transferred to decrease proportionally with the inverse square of the distance from the source. Integrated over the time window necessary to observe a depolarization in the receiving neurons, this mechanism predicts a drop in assembly-spread velocity proportional to the inverse of the distance. Wave-like patterns have been observed throughout the brain, though so far mostly attributed to feed-forward networks (Meijer and Coombes, 2014): we found that the velocity profile as a function of distance for assemblies is consistent with a spatiotemporal spread due to self-sustained electromagnetic waves (**Figure 2C**).

The mechanism by which radiation can excite neuronal activity, to the point of remotely triggering action potentials, is not unfamiliar (Huang et al., 2010); the most obvious candidate vehicle to host the magnetic field outside the neuron are glial cells, and/or the extracellular matrix: packed with ions, they are available in large quantities and continuously surrounding the network of neurons, providing a coherent medium where electromagnetic waves can propagate self-substainedly. Indeed, the cooperative action of neurons and glia has already been proven capable of influencing the timing of neuronal activity (Anastassiou et al., 2010).

In conclusion, disparate empirical findings both *in vivo* and *in vitro* could most readily be accommodated theoretically in the integration of three distinct signaling mechanisms over an epoch of some 250–300 ms (**Figure 2D**). As such, this approach to analyzing brain operations at the meso-scale would have the potential for a more accurate modeling of drug action and more generally a quantification of holistic brain states with a temporal and spatial resolution commensurate with neurophysiological and neurochemical events.

## Author Contributions

A-SB is responsible for the data of **Figure 1** and presentation of **Figures 1** and **2**; FF is responsible for the data of **Figure 2**. SG provided the original idea as well as background material. All authors contributed to the preparation, writing, and proof-reading of the manuscript.

## Conflict of Interest Statement

The authors declare that the research was conducted in the absence of any commercial or financial relationships that could be construed as a potential conflict of interest.

The reviewers NM and AS and handling Editor declared their shared affiliation, and the handling Editor states that the process nevertheless met the standards of a fair and objective review.
