# Supplementary material for: The Features and Functions of Neuronal Assemblies: Possible Dependency on Mechanisms beyond Synaptic Transmission
Source: Front Neural Circuits. 2017 Jan 10;10:114. doi: 10.3389/fncir.2016.00114 (PMC5223595; doi:10.3389/fncir.2016.00114)
Supplement: Supplementary file 1 [file Data_Sheet_1.docx]

Supplementary Material

**NEURONAL ASSEMBLIES: THEIR FEATURES AND FUNCTIONS DEPEND ON NON-CLASSICAL MECHANISMS**

Antoine-Scott Badin*, Francesco Fermani, Susan Greenfield

*** Correspondence:** Antoine-Scott Badin: [scott.badin@neuro-bio.com](mailto:scott.badin@neuro-bio.com)

# Supplementary materials and methods

1. *Experimental procedures for dissection and slicing of functional brain slices*:

Wistar p14 (parturition day 14) male rats were anaesthetized with Isoflurane before dislocating the neck, in accordance with the Home Office Schedule 1 culling. The brain was then quickly extracted in ice-cold artificial cerebrospinal fluid (aCSF) and prepared for slicingas previously described ([Badin et al., 2013](#_ENREF_3); [Badin et al., 2016](#_ENREF_4)). Two types of slices were acquired: either containing thalamus (VPM nucleus) and somatosensory cortex (SEN) as previously described ([Agmon and Connors, 1991](#_ENREF_1)) with intact thalamocortical connections, or simple coronal slices through the agranular insular cortex (PFC) or primary somatosensory cortex (SEN) for direct stimulation experiments. In all case, slices were 400 μm-thick using a vibratome (Leica Microsystems), and left to recuperate at room temperature for a minimum of 1 hour. Following this, slices were stained with the red VSD di-4-ANEPPS (7% Cremophore EL in DMSO containing a final di-4-ANEPPS concentration of 350 μM) for 30 minutes, before being washed and left to recuperate at room temperature for a further 45 minutes before experimentation.

For recording of two-dimensional patterns of activity, assemblies were triggered using a concentric bipolar electrode with 25 μm tip-width placed on the slice in the location of interest (either VPM in thalamocortical slices, LII/III in slices containing agranular insular cortex, i.e. PFC, see Figure 1, of LIV in slices containing SEN) to carry out one of two types of experiments: either direct stimulations applied to the cortex (PFC or SEN) with 30 Volts pulses (0.1 ms in duration), or applied (in thalamocortical slices) to the VPM nucleus of the thalamus, with a 0.1 ms 60 Volts pulse, for neuronal activation of the SEN. Data were acquired, processed and analysed using Matlab and a VSDI toolbox specifically designed to rasterising and averaging VSDI data ([Bourgeois et al., 2014](#_ENREF_5)), as described previously ([Badin et al., 2013](#_ENREF_3); [Badin et al., 2016](#_ENREF_4)).

1. *Modelling of neuronal population activity*

*Synaptic transmission in a network:*

We describe the activity of the single cell via the exponential integrate-and-fire (EIF) neuron model ([Fourcaud-Trocme et al., 2003](#_ENREF_6)), which has been shown empirically to capture the voltage dynamics of a variety of classes of cortical neurons ([Badel et al., 2008](#_ENREF_2); [Harrison et al., 2015](#_ENREF_8)) for its accurate description of the membrane voltage dynamics that accounts for the exponential sodium-channel activation. The network dynamics is then derived in a bottom-up fashion: neurons receive both external inputs (for the cortex, mostly via the thalamus) and feedback from neighbouring neurons. The probability to make a connection (synapse) decays with distance and the length scale of such decay has been measured experimentally with increasing accuracy ([Hellwig, 2000](#_ENREF_9); [Perin et al., 2011](#_ENREF_11)); this results in the effective contribution of neurons in the network having to be weighted by their distance to the post-synaptic neuron. The sub-threshold voltage dynamics at each point is therefore ([Jirsa and Haken, 1996](#_ENREF_10)):

$$\tau_{m}\frac{\partial V}{\partial t}=E-V+\Delta_{T}e^{\frac{V-V_{T}}{\Delta_{T}}}+\sqrt{2 \tau_{m}}\sigma_{V}\xi\left( t \right)+\tau_{m}a \sum_{l} \sum_{t_{l}} \delta\left( t-t_{l}^{k}-\tau_{d}^{l}\left( x \right) \right)\rho(x-x_{l})$$

where $\tau_{m}$ is the membrane time-constant, $\sigma_{V}$ the fluctuations amplitude in synaptic input, $\xi\left( t \right)$ Gaussian noise, a is the post-synaptic potential (PSP) amplitude, $t_{l}^{k}$the spike time of the pre-synaptic neuron l, $\tau_{d}^{l}$ the physiological time-delay due to synaptic delay and finite propagation speed (c) and $\rho$ is the connectivity kernel.

The model above has been extended in several directions to include phenomena like adaptation or synaptic dynamics ([Gerstner and Kistler, 2002](#_ENREF_7)): for the scope of the present work we limit at its basic formulation, which still retains the dominant network dynamics and eases the disentangling exercise we focus on in this work.

*Diffusion of neuromodulators:*

The spread of bioactive agents in the brain tissue can be modelled with a multidimensional diffusion equation:

$$\frac{\partial\phi}{\partial t}=D \nabla^{2}\phi+S(x,t)$$

where $\phi$is the concentration, D the diffusion coefficient and S accounts for distribution of sources and sinks in space and time. The diffusion coefficient encodes information on the tortuosity of the tissue and the easiness that a particular molecule has in diffusing in the surrounding space. The sources are the locations where the molecule is secreted and the sinks where is metabolised.

*Electromagnetic transmission:*

A time-varying electric field induces a magnetic field and viceversa, cycle that can eventually lead to self-sustained electromagnetic waves. By energy conservation, the intensity delivered scales with the inverse square of the distance ($r$) from the source of the time-varying field:

$$p=\frac{\kappa}{r^{2}}$$

and $\kappa$ is a constant. The membrane potential depolarization is then a function of the intensity received, to first order equal to $f\left( p \right)\sim\alpha p$, where $\alpha$ is a constant. For the membrane potential to experience a net depolarization $\Delta_{V}$, packages of energy have to accumulate over time:

$$\int_{0}^{t} dt^{'}\alpha pf=\Delta_{V}$$

where $f$ is the frequency of the electromagnetic wave. Integrating and rearranging the equation above, one can compute the velocity profile of the activity spread:

$$v_{\mathrm{dep}}= \frac{1}{r}\left( \frac{f\alpha\kappa}{\Delta_{V}} \right)$$

This profile can be directly compared with the depolarization spread of neuronal assemblies and the comparison informs on whether this mechanism is any relevant for the dynamics and the scale under investigation.

# Supplementary references

Agmon, A., and B.W. Connors. (1991). Thalamocortical responses of mouse somatosensory (barrel) cortex in vitro. *Neuroscience*. 41:365-379.

Badel, L., S. Lefort, R. Brette, C.C. Petersen, W. Gerstner, and M.J. Richardson. (2008). Dynamic I-V curves are reliable predictors of naturalistic pyramidal-neuron voltage traces. *Journal of neurophysiology*. 99:656-666.

Badin, A.S., J. Eraifej, and S. Greenfield. (2013). High-resolution spatio-temporal bioactivity of a novel peptide revealed by optical imaging in rat orbitofrontal cortex in vitro: Possible implications for neurodegenerative diseases. *Neuropharmacology*. 73C:10-18.

Badin, A.S., P. Morrill, I.M. Devonshire, and S.A. Greenfield. (2016). (II) Physiological profiling of an endogenous peptide in the basal forebrain: Age-related bioactivity and blockade with a novel modulator. *Neuropharmacology*. 105:47-60.

Bourgeois, E.B., B.N. Johnson, A.J. McCoy, L. Trippa, A.S. Cohen, and E.D. Marsh. (2014). A toolbox for spatiotemporal analysis of voltage-sensitive dye imaging data in brain slices. *PLoS One*. 9:e108686.

Fourcaud-Trocme, N., D. Hansel, C. van Vreeswijk, and N. Brunel. (2003). How spike generation mechanisms determine the neuronal response to fluctuating inputs. *J Neurosci*. 23:11628-11640.

Gerstner, W., and W. Kistler. (2002). Spiking Neuron Models. Cambridge University Press, Cambridge.

Harrison, P.M., L. Badel, M.J. Wall, and M.J. Richardson. (2015). Experimentally Verified Parameter Sets for Modelling Heterogeneous Neocortical Pyramidal-Cell Populations. *PLoS computational biology*. 11:e1004165.

Hellwig, B. (2000). A quantitative analysis of the local connectivity between pyramidal neurons in layers 2/3 of the rat visual cortex. *Biological cybernetics*. 82:111-121.

Jirsa, V.K., and H. Haken. (1996). Field Theory of Electromagnetic Brain Activity. *Phys Rev Lett*. 77:960-963.

Perin, R., T.K. Berger, and H. Markram. (2011). A synaptic organizing principle for cortical neuronal groups. *Proc Natl Acad Sci U S A*. 108:5419-5424.
